# The effect of alpha lipoic acid and caffeine loaded into chitosan nanoparticles on the hypothalamic amino acid neurotransmitters in a rat model of obesity

**DOI:** 10.1038/s41598-025-27087-6

**Published:** 2025-11-27

**Authors:** Yasser A. Khadrawy, Noha M. Khoder, Mayada M. El-Gizawy, Eman N. Hosny, Hussein G. Sawie, Nourhan S. Elkholy, Hanaa M. Amer, Heba S. Aboul Ezz

**Affiliations:** 1 Medical Physiology Department, Medical Research and Clinical Studies Institute, National Research Centre, Giza, Egypt; 2 Nawah Scientific Co ., Cairo, Egypt; 3 Radiology and Medical Imaging Technology Department, Faculty of Applied Health Sciences Technology, Nile Valley University , Fayoum, Egypt; 4 Faculty of Science , Cairo University , Cairo, Egypt

**Keywords:** Obesity, Hypothalamus, AAs, Leptin, Ghrelin, α-LA, Caf-CNPs, Biochemistry, Neuroscience, Physiology

## Abstract

The present study was carried out to investigate the physiological role of the hypothalamic amino acid neurotransmitters (AAs) in feeding behavior and obesity. Caffeine loaded into chitosan nanoparticles (Caf-CNPs) and/or α-lipoic acid (α-LA) were used to investigate their therapeutic effects on the changes in hypothalamic glutamine, glutamate, aspartate, GABA, glycine and taurine and serum levels of leptin and ghrelin in addition to the body mass index (BMI) of obese rats. Rats were divided into lean control group, rat model of obesity induced by high fat diet (HFD) group (obese control), and rat model of obesity group treated with Caf-CNPs and /or α-LA. Obese rats showed a higher value of BMI, elevated levels of leptin, reduced levels of ghrelin, and increased levels of hypothalamic glutamate, aspartate, GABA and taurine. Treatment with Caf-CNPs improved BMI and didn’t affect the changes in hypothalamic AAs, leptin and ghrelin induced in obese rats. Treatment with α-LA improved BMI, leptin and ghrelin levels and restored the changes in AAs. Co-administration of Caf-CNPs and α-LA restored BMI, leptin, ghrelin, aspartate and taurine to control-like values, however, there were elevated levels of glutamine, glutamate, GABA and glycine. The present study showed the substantial role of hypothalamic AAs in controlling feeding behavior and development of obesity. It may be concluded that Caf-CNPs and/or α-LA could potentially act as anti-obesity agents.

## Introduction

Globally, the abrupt increase in the number of obese and overweight people, especially in recent years, causes obesity to be considered a universal epidemic disease. The report of the World Health Organization (WHO, 2024) recorded over one billion adults as overweight and classified 890 million people as clinically obese^1,2^. In addition to being linked to a higher overall mortality rate, obesity increases the risk of numerous comorbidities, such as type 2 diabetes mellitus, cancer^3^, cardiovascular diseases^4^, and musculoskeletal disorders^5^. These raise an urgent need to treat obesity and prevent the development of these comorbidities.

Obesity is viewed as a chronic complex disease caused by excessive fat deposits that can impair health. Mathematically, obesity is regarded as an imbalance between the intake and expenditure of energy^6^ that is regulated by central circuits controlling food intake and energy homeostasis^7^. Disturbances in these circuits are critical in body weight and adiposity^8^. Impairment in the metabolism of neurotransmitters communicating these circuits has been found to underlie the pathogenesis of obesity. Numerous neurotransmitters, such as serotonin, dopamine, glutamate and GABA, are involved in energy homeostasis through regulating food intake and/or energy expenditure and are altered in obesity. Therefore, restoring these changes may be pharmacologically targeted to control obesity^9^.

Glucose is the primary energy substrate for the brain and supports neurotransmission; any disruptions in glucose metabolism may have a direct effect on brain function through a mechanism that is independent of environmental stressors^10^. In this context, alterations in amino acid neurotransmitter homeostasis were reported in obese and diabetic rats due to impaired brain glucose metabolism^11^.

The hypothalamus is the most important brain region that contains a heterogeneity of neuronal populations regulating energy homeostasis through feeding and energy expenditure^12^. It contains specialized neuronal populations that monitor the peripheral metabolic state and regulate food intake and energy metabolism. These neurons include the lateral hypothalamus (LH)^13^, ventromedial hypothalamus^14^, paraventricular nucleus and arcuate (ARC) nucleus^15,16^. In addition, pro-opiomelanocortin (POMC) neurons near the median eminence and third ventricle can detect and respond to circulating hormones and nutrient signals, including insulin^17^, leptin^18^, ghrelin^19^, glucagon-like peptide-1^20^, glucose^21^ and fatty acids^22^.

Major neurotransmitters present in the hypothalamus are glutamate and γ-aminobutyric acid (GABA)^23^. The injection of glutamate, or its excitatory agonists, NMDA, AMPA, and kainic acid intracerebroventricularly^24^, or in the lateral hypothalamus^25^, provokes an intensive food intake in rats. POMC neurons can communicate through amino acid transmitters. Glutamatergic and GABAergic cells are present in comparably significant numbers among POMC neurons and play a crucial function in regulating energy balance^26,27^. Moreover, it has been reported that chronic activation of GABAergic neuronal populations located in the median region of the arcuate hypothalamus^27^ promotes feeding and obesity^28^. On the other hand, brain taurine is known as a neuromodulator and osmoregulatory agent and participates in other functions, such as modulating neuronal excitability, controlling the cardiorespiratory system, regulating appetite, and acting as antioxidant^29^.

The consumption of dietary fats has been found to induce apoptosis of neurons and reduce synaptic inputs in the ARC and LH neurons in rodents^30^. Thus, more research into the pathophysiology of hypothalamic obesity may improve our understanding of the processes underlying obesity and weight regulation and result in the creation of novel therapeutic approaches.

Leptin is a satiety hormone secreted by white adipocytes and acts by negative feedback control on the hypothalamus to regulate appetite and expenditure of energy^31^. Ghrelin, known as the hunger hormone, is formed mainly in the X/A cells of the gastric fundus and acts on the hypothalamus to stimulate food intake and promote weight gain by fat accumulation and adiposity^32^. Leptin and ghrelin work together to regulate hunger and satiety sensations by signaling different nuclei within the hypothalamus. An imbalance or dysregulation of any or both of these hormones may have an adverse effect on the body’s energy homeostasis^33^.

Caffeine is a methylxanthine compound found in coffee, tea, and chocolate and is widely consumed all over the world. Caffeine has been used as a fat burner and appetite suppressor for weight loss^34^. It also induced an anti-adipogenic effect via inhibition of PPARγ2 and C/EBPα, key genes in adipogenesis^35^. In addition, caffeine can reduce obesity by modulating metabolism and antioxidant and anti-inflammatory effects^36^. Caffeine can also modulate obesity by acting on the intestinal microbiota and its metabolites^37^. Our previous data showed that caffeine loaded into chitosan nanoparticles reduced the BMI of obese rats^38^.

Alpha lipoic acid (α-LA) is a natural sulfur-containing coenzyme synthesized in the mitochondria and plays an important role in energy metabolism^39^. α-LA acts as an anti-obesity agent. It reduces the body weight and BMI^38,40^. Its anti-obesity effects are mediated by the inhibition of hypothalamic 5´-adenosine monophosphate (AMP)-activated protein kinase activity, an enzyme that is critical in the regulation of food intake and energy expenditure^41^.

The present study investigates the central anti-obesity effects of caffeine-loaded into chitosan nanoparticles and/or alpha lipoic acid through their effects on body mass index and the hypothalamic amino acid neurotransmitters glutamate, aspartate, GABA, glycine and taurine. In addition, their effects on the serum levels of leptin and ghrelin in obese rats were examined.

## Materials and methods

### Chemicals

α-Lipoic acid (α-LA) was obtained from EVA Pharma for Pharmaceuticals and Medical Appliances, Cairo, Egypt. Chitosan and caffeine were purchased from Sigma-Aldrich, Germany. Methanol and sodium tripolyphosphate were purchased from Loba Chemie PVT, LTD (Mumbai, India); low-MW chitosan and trifluoroacetic acid were supplied by Sigma-Aldrich (Saint Louis, USA); and acetonitrile from Waters Associates, Inc. (Milford, Massachusetts, USA). Caf-CNPs (20 mg/kg) were prepared daily by dissolving 100 mg of Caf-CNPs in 10 ml saline solution (0.9%). α-LA (100 mg/kg) was prepared by dissolving 400 mg in 10 ml of saline solution (0.9%).

### Preparation of caffeine-loaded into chitosan nanoparticles (Caf-CNPS)

The preparation of caffeine-loaded into chitosan nanoparticles (Caf-CNPs) was performed according to the procedure of Sahudin et al.^42^. The gelation of chitosan (CS) solutions was induced with the crosslinking compound sodium tripolyphosphate (TPP) (0.1% in distilled water), while low molecular weight chitosan (0.2%) was dissolved in 1% acetic acid. The addition of TPP into the CS solution resulted in the spontaneous formation of CS NPs. TPP-containing caffeine (0.1%) was then added dropwise into 25 ml of CS solution while stirring for 30 min with a magnetic stirrer (1000 rpm) at room temperature to form Caf-CNPs. The nanoparticles were ultrasonicated by an ultrasonic homogenizer (model VCX 500, frequency 20 kHz, DIAGGE ILL 60061, USA, with titanium probe 50% amplitude with a 90% pulse) for a few minutes and then separated from their suspension by centrifugation at 10,000 rpm for 30 min.

### Characterization of Caf-CNPs

#### Transmission electron microscopy (TEM) imaging

Imaging by TEM was used to determine the shape and average size of the Caf-CNPs. One mg/ml of Caf-CNPs solution was fixed on the surface of a TEM grid and incubated for a few minutes. The grid surface was dried with filter paper to remove the excess fluid and then air dried at room temperature. It was then uploaded into the transmission electron microscope (JEM-HR-2100 electron microscope, Japan) to photograph the nanoparticles at a total magnification of 6.00 kx with accelerating voltage 200 kV.

#### Zeta sizer and zeta potential measurement

The mean particle size, zeta potential, and size distribution of freshly prepared Caf-CNPs were estimated by dynamic light scattering with the help of a particle sizing system which consisted of a zeta sizer (Nano ZS, Malvern, UK) at 25 °C. The average of three independent measurements was calculated and included in the data.

#### Encapsulation efficiency

Encapsulation efficiency (EE) was measured using the Nanosep technique. Half a ml of the formula was placed on a centrifugal filter unit (Amicon Ultra-10 K, Merk Millipore, Tullagreen, Carrigtwohill), then centrifuged at 6000 rpm for 15 min at a temperature of 4 °C. The supernatant was used for testing the unentrapped drug.

The resulting supernatant was filtered and analyzed using an HPLC validated method.

$${\text{EE\% }} = \:\frac{{{\text{Total}}\:{\text{amount}}\:{\text{of}}\:{\text{caffeine}} - {\text{unentrapped}}\:{\text{caffeine}}\:{\text{in}}\:{\text{supernatant}}}}{{{\text{total}}\:{\text{amount}}\:{\text{of}}\:{\text{caffeine}}}}\: \times \:100$$

#### In vitro release studies

The in vitro release study was done using the dialysis sac technique. Phosphate- buffered saline (PBS) pH 7.4 was prepared and kept at 37 °C. 1 ml of Caf-CNPs was inserted in a dialysis bag (12–14 kDa cut off, Sigma Aldrich, Germany), which was sealed from top and bottom and immersed into 100 ml PBS for 10 h in tightly closed flasks. The whole system was positioned in a shaking incubator (Jeio tech SI-300, Seoul, Korea) at 37 °C and adjusted to 100 rpm rotation. At predetermined intervals, 2 ml of the sample were removed from the release medium and replaced immediately with 2 ml of warm fresh buffer. The removed samples were applied to the HPLC and analyzed as described.

$$\:{\text{Release rate }} = \frac{{{\text{Released}}\:{\text{amount}}\:{\text{of}}\:{\text{caffeine}}}}{{{\text{total}}\:{\text{amount}}\:{\text{of}}\:{\text{caffeine}}}}\: \times \:100$$

### High-performance liquid chromatography (HPLC)

The caffeine content, encapsulation efficiency, and release were measured quantitatively using an HPLC system equipped with Waters 2695 LC, 996 PDA detector, a low-pressure mixing pump, an auto-sampler with a 100 µl sample loop, and a 5 μm reversed-phase Kromasil Cyano (CN) column (250 × 4.6 mm). The mobile phase consisted of (A) 0.1% trifluoroacetic acid (TFA) and deionized water and (B) acetonitrile. The gradient began using 5% B at 0 min and was raised to 8% A at 4 min. The solvent B was increased from 8% to 50% between 4 and 5 min, held at 50% until 7 min, then decreased back to 5% by 8 min. The system was re-equilibrated at initial conditions (5% B) from 8 to 11 min. The flow rate was 1.5 ml/min, and the chromatogram was monitored at 270 nm.

### Experimental animals

Fifty healthy adult male Wistar albino rats (12–14 weeks old) were purchased from the animal house of the National Research Centre, Giza, Egypt. The weights of the rats ranged from 150 to 170 g. Animals were distributed in plastic cages with stainless steel covers (eight rats per cage). Rats were left for 7 days to adapt to the laboratory conditions and allowed free access to tap water and food. Animals were placed in a controlled temperature (20–25 °C) and humidity (50%−60%), and artificially illuminated (12 h dark/light cycle) room that was free of any contamination. The experimental protocol was approved by the Ethics Committee of the National Research Centre (approval number 20149) in accordance with ARRIVE guidelines.

### Induction of rat model of obesity

The induction of the rat model of obesity was carried out by feeding rats on a high fat-diet (HFD) consisting of 45% fat, 41% carbohydrate, and 19% protein for 20 weeks, according to McNeilly et al.^43^. After 20 weeks, the body mass index (BMI) was calculated using the formula:

$${\text{BMI }} = \frac{{{\text{Body~weight~(g)}}}}{{{\text{Body~length}}^{{\text{2}}} {\text{~(cm}}^{{\text{2}}} {\text{)}}}}$$

The BMI was used as a marker for obesity. Rats having a BMI greater than 0.68 g/cm^2^ were considered obese and were used for the experiment according to Novelli et al.^44^ while rats with a BMI less than 0.68 g/cm^2^ were discarded.

### Experimental design

At the beginning of the experiment, fifty rats were divided into two groups: lean control rats and rat model of obesity (obese control). In the lean control group, ten rats were fed a standard diet of 2% fat, 67% carbohydrate, and 21% protein throughout the experimental period. The rest of the animals (40 rats) were fed on HFD to obtain the rat model of obesity as described by McNeilly et al.^43^. These animals were divided into four groups. The first group represented the rat model of obesity (obese control) and was treated daily with saline solution for 4 weeks. The second group was the rat model of obesity treated by gavage daily with α-LA (100 mg/kg)^45^ for 4 weeks. Each rat received 2.5 ml/kg orally. The third rat model of obesity group was treated intraperitoneally with Caf-CNPs (20 mg/kg)^46^ daily for 4 weeks. Each rat received an i.p. injection at a volume of 2 ml/kg. Finally, the fourth group consisted of a rat model of obesity treated daily with a combination of α-LA and Caf-CNPs with a one-hour interval in-between for 4 weeks.

At the end of the experimental period, blood samples were withdrawn from the retro-orbital venous plexus of the rats and collected in clean heparinized capillary tubes. The blood samples were centrifuged at 3000 rpm for 15 min at 4 °C to separate sera, which were stored at −80 °C till the measurement of leptin and ghrelin levels. Then the animals were sacrificed, and the brain of each rat was removed out and dissected on an ice-cold Petri dish to obtain the hypothalamus. The brain area of each rat was weighed and frozen at ‒80 ^○^C until the analysis of the amino acid neurotransmitters glutamine, glutamate, aspartate, GABA, glycine and taurine by HPLC. In the present study, rats were sacrificed by sudden decapitation without anesthesia to avoid its effects on amino acid neurotransmitters.

The following table summarizes the experimental design.

**Table:** 

Animal groups	20 weeks	Daily treatment for 4 weeks
Lean control rats	Fed a standard diet	Saline (0.9%)
Obese control rats	Fed HFD	Saline (0.9%)
Obese control rats treated with α-LA	Fed HFD	α-LA (100 mg/kg)
Obese control rats treated with Caf-CNPs	Fed HFD	Caf-CNPs (20 mg/kg)
Obese control rats treated with α-LA + Caf-CNPs	Fed HFD	α-LA + Caf-CNPs
	

### Determination of amino acid neurotransmitters

The amino acid neurotransmitters glutamate, aspartate, glutamine, GABA, glycine and taurine were determined quantitatively in the hypothalamus according to Márquez et al.^47^. The HPLC system consisted of a quaternary pump (YL9110), vacuum degasser (YL9101), LC autosampler (YL9150), UV/visible detector (YL9120), column compartment (YL9131) (Young in Chromass, Republic of Korea) and Water 5 μm C-18 reversed phase column (5 μm particle size, 150 × 4.6 mm I.D., Japan). The mobile phase consisted of methanol/water 70/30 (v/v), containing 0.6% glacial acetic acid and 0.008% triethylamine. The amino acids were detected at 254 nm using a UV detector. The amino acid concentrations were expressed as µmol/g.

### Determination of serum levels of leptin and Ghrelin

The serum levels of leptin and ghrelin were determined by ELISA kits. Rat ELISA kits of leptin (Catalogue No. SG-20057) and ghrelin (Catalogue No. SG-20996) were obtained from Sino Gene Clon Biotech Co., Ltd (Hang Zhou, China).

### Statistical analysis

The present data were checked for the normality of distribution using visual inspection of Q–Q plots and box plots, the Shapiro-Wilk test and measures of skewness and kurtosis. In the Shapiro-Wilk test, all the p-values for all tested groups were above 0.05, indicating that our data followed a normal distribution. Also, all Ζ-values of the skewness and kurtosis were within the ± 1.96 range for a normal distribution. The visual inspection of Q–Q plots and box plots showed that the present data were normally distributed for all groups.

The statistical difference between groups was determined by one-way analysis of variance (ANOVA) using the Statistical Package for Social Sciences (SPSS) program. When the difference was statistically significant (p-value < 0.05), the Duncan post hoc test was applied, and the data were expressed as means ± S.E.M.

### Ethics approval

Animal procedures were approved by the Ethics Committee of the National Research Centre (with ethical approval number of 20149) and were performed in compliance with the recommendations of the National Institutes of Health Guide for Care and Use of Laboratory Animals (publication no. 85–23, revised 1985) in accordance with ARRIVE guidelines.

## Results

### Transmission electron microscopy (TEM) imaging of Caf-CNPs

**Fig. 1:**
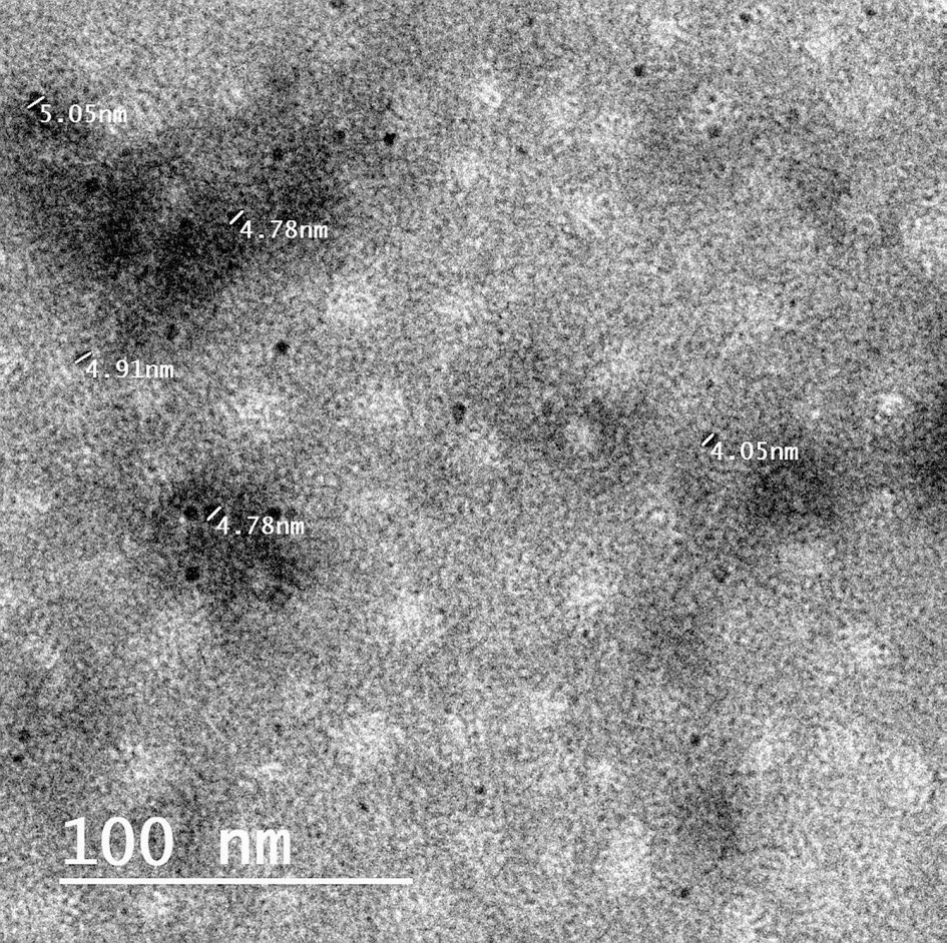
TEM image of Caf-CNPS.

TEM imaging revealed that Caf-CNPs have a spherical shape, and their sizes ranged from 4.05 to 5.05 nm (Fig. 1).

### Particle size and zeta potential

**Fig. 2:**
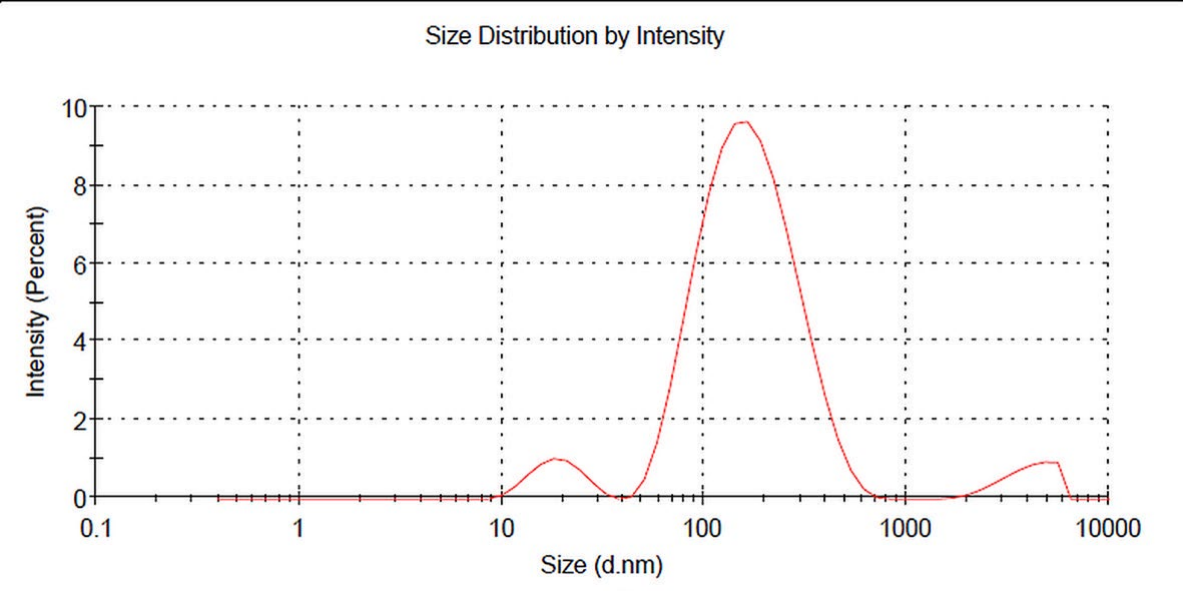
Size distribution of Caf-CNPs.

As shown in Fig. 2, the calculated hydrodynamic particle size of Caf-CNPs was 122.76 ± 2.141. The polydispersity index distribution (PDI) for Caf-CNPs was 0.441.

**Fig. 3:**
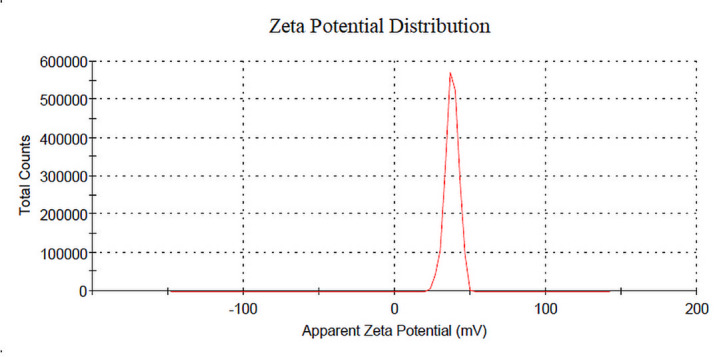
Zeta potential of Caf-CNPs.

The zeta potential of Caf-CNPs had a range of + 32.4 mV to + 39.9 mV (Fig. 3). This value of the potential prevents the nanoparticles from agglomerating and provides them with increased stability.

### Encapsulation efficiency and in vitro release

**Fig. 4:**
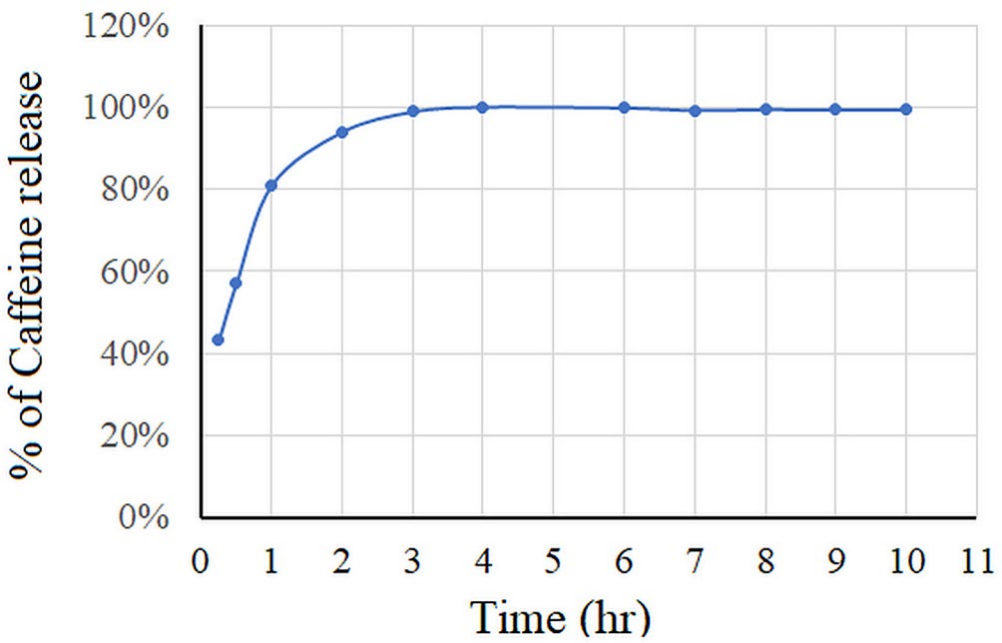
Rate of caffeine release from chitosan.

The average encapsulation efficiency of chitosan nanoparticles for caffeine was 34.18 ± 4.579%. The in vitro release study revealed that the rate of caffeine release was 60% after 30 min, 80% after one hour and 100% after 3 h. The results indicated a sustained release of caffeine that extended over 10 h (Fig. 4).

### BMI results

The present data revealed that the BMI of lean control rats was 0.54 g/cm^2^, which is below the reported limit of the BMI of obese rats (> 0.68 g/cm^2^). The BMI of the obese control rat model was 0.77 g/cm^2^, which is greater than the reported BMI of obese rats.

ANOVA revealed a significant difference in BMI between the different groups. A significant increase was recorded in the BMI of obese control rats (*p* = 0.001, F (4,45) = 36.435) as compared to lean control rats. Treatment with Caf-NPs reduced the BMI to 0.65 g/cm^2^. Treatment with α-LA reduced the BMI to 0.64 g/cm^2^. Co-treatment with Caf-NPs and α-LA reduced the BMI to 0.62 g/cm^2^ (Table 1).

As shown in Table 2, the BMI of lean control rats was 0.514 g/cm^2^ after 20 weeks of feeding on a standard diet and 0.54 g/cm^2^ after 24 weeks. Obese rats’ BMI was 0.73 g/cm^2^ after 20 weeks of feeding HFD and 0.771 g/cm^2^ after 24 weeks. The BMI of obese rats (second group) was 0.787 g/cm^2^ after 20 weeks of HFD feeding and 0.638 g/cm^2^ after 4 weeks of daily treatment with α-LA. Obese rats of the third group had a BMI of 0.79 g/cm^2^ after 20 weeks of HFD feeding and 0.649 g/cm^2^ after 4 weeks of daily treatment with Caf-CNPs. The BMI of obese rats of the fourth group was 0.791 g/cm^2^ after 20 weeks of HFD feeding and 0.638 g/cm^2^ after 4 weeks of daily treatment with α-LA + Caf-CNPs.

### Excitatory amino acid neurotransmitters and metabolites

**Fig. 5:**
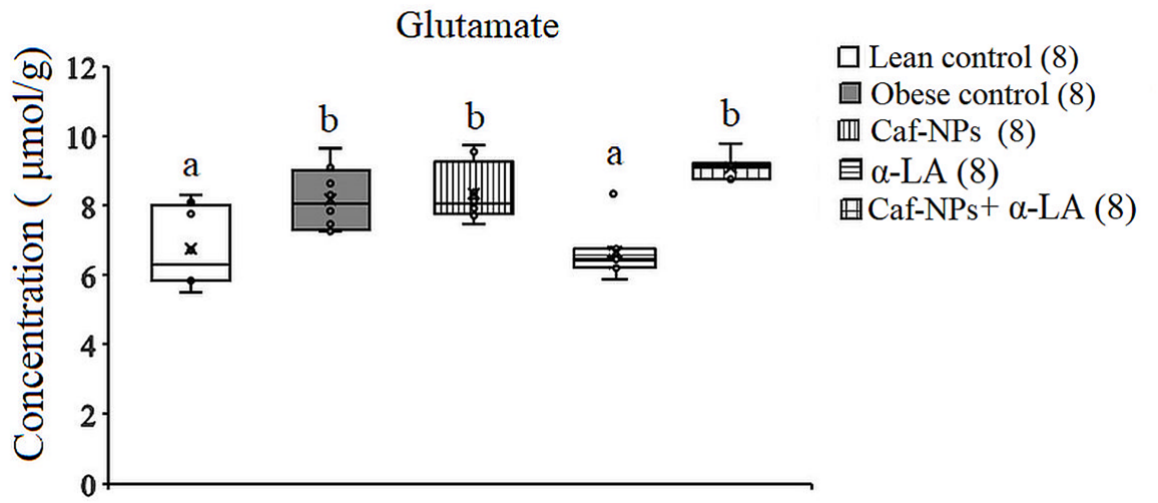
Effect of Caf-CNPs and/or α-LA on the level of glutamine (µmol/g) in the hypothalamus of rat model of obesity.

**Fig. 6:**
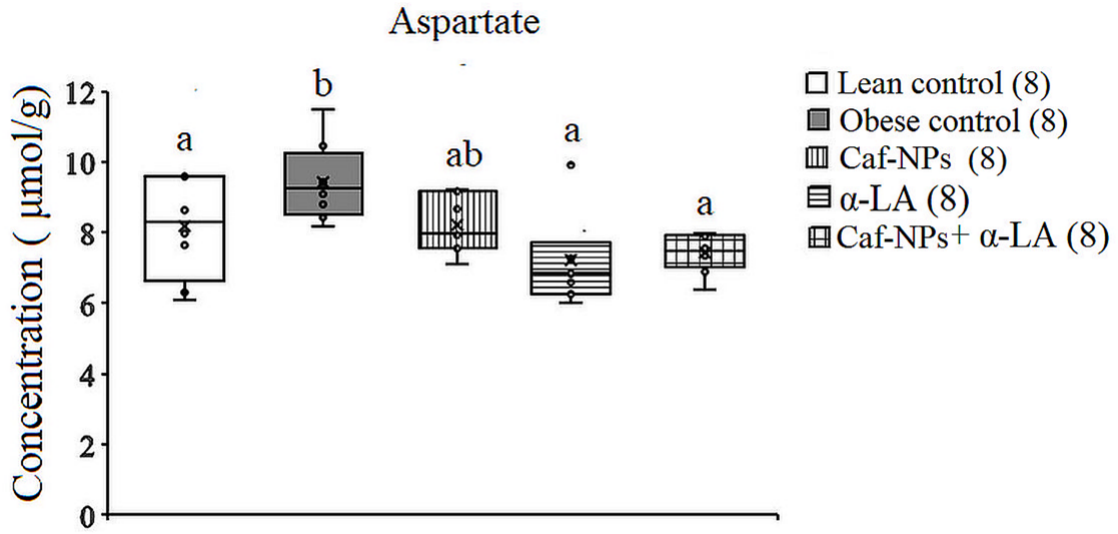
Effect of Caf-CNPs and/or α-LA on the level of glutamate (µmol/g) in the hypothalamus of rat model of obesity.

**Fig. 7:**
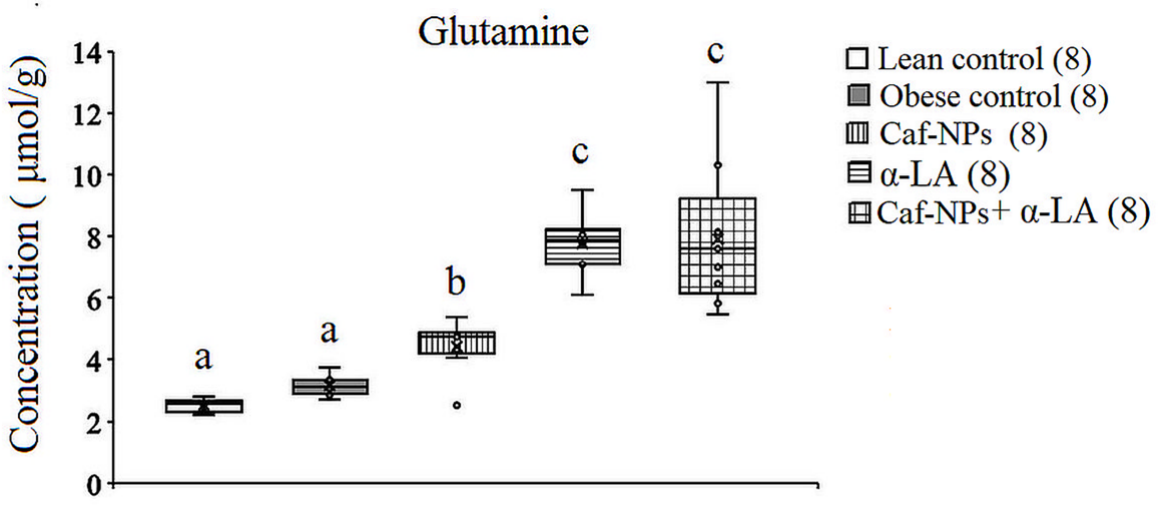
Effect of Caf-CNPs and/or α-LA on the level of aspartate (µmol/g) in the hypothalamus of rat model of obesity.

In the hypothalamus of obese rats, ANOVA revealed significant differences in glutamate and aspartate between groups. A significant increase in glutamate (*p* = 0.001, F (4,35) = 7.792) and aspartate (*p* = 0.009, F (4,35) = 4.146) was obtained in obese control, recording 19.2% and 18.2%, respectively, as compared to lean control rats. Treatment of obese control rats with Caf-CNPs increased glutamate levels (21.6%) significantly and improved aspartate (−3.1%) levels, while α-LA restored the levels of glutamate and aspartate to control-like values, recording − 1.6% and − 8.9%, respectively. When obese control rats were treated with a combination of Caf-CNPs and α-LA, the significant increase in glutamate (32.6%) was maintained, whereas aspartate (−7.1%) levels returned to lean control values (Figs. 5 and 6). Glutamine levels showed a nonsignificant increase in obese control rats but increased significantly (*p* = 0.001, F (4,35) = 25.007) after treatment with Caf-NPs, α-LA or their combination, recording 75.3%, 203.3% and 171.62%, respectively (Fig. 7).

### Inhibitory amino acid neurotransmitters

**Fig. 8:**
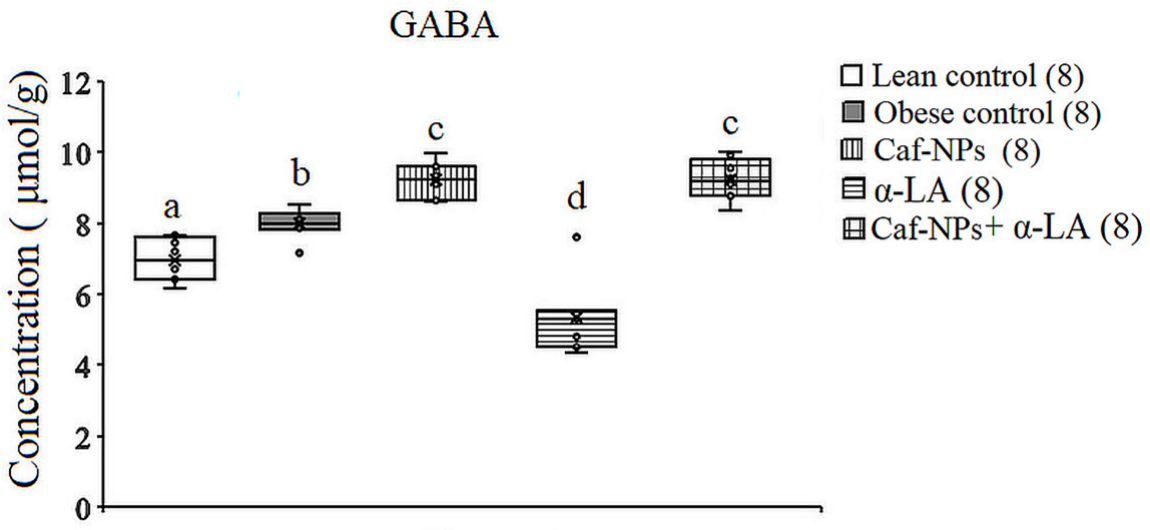
Effect of Caf-CNPs and/or α-LA on the level of GABA (µmol/g) in the hypothalamus of rat model of obesity.

**Fig. 9:**
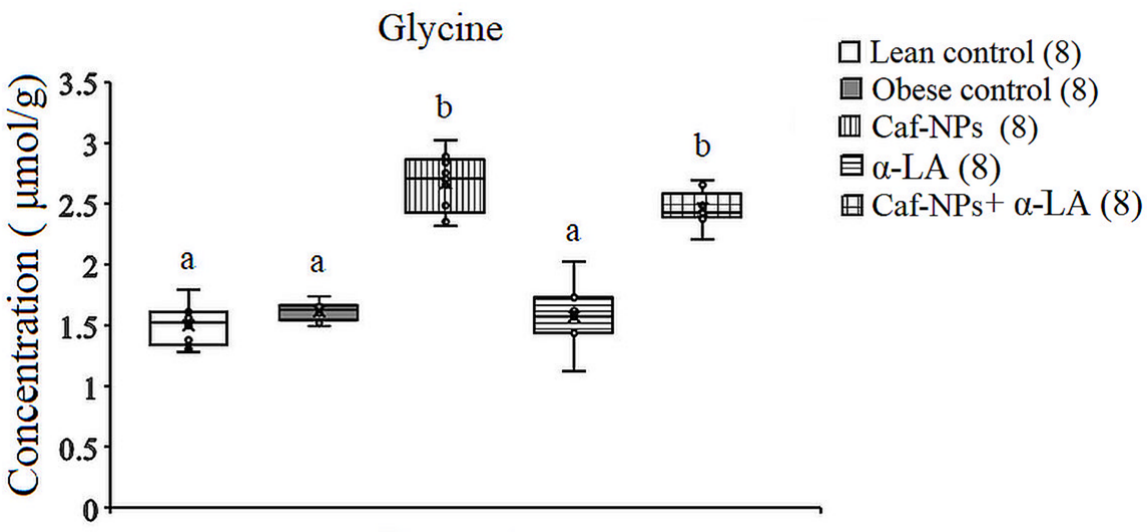
Effect of Caf-CNPs and/or α-LA on the level of glycine (µmol/g) in the hypothalamus of rat model of obesity.

The hypothalamic GABA levels increased significantly (*p* = 0.001, F (4, 35) = 30.627) in obese control rats, recording 14.61% as compared to lean control rats. α-LA reversed the increase in GABA to a significant decrease, recording − 23.73% as compared to the lean control. However, in obese control rats treated with Caf-CNPs alone or in combination with α-LA, GABA increased significantly by 32.3% and 34.3%, respectively, above the lean control (Fig. 8). Glycine didn’t change in obese control rats (7.7%) or after treatment with α-LA (3.1%). However, glycine increased significantly (*p* = 0.001, F (4,35) = 71.855) when obese control rats were treated with Caf-CNPs (77.7%) alone or combined with α-LA (63.7%) (Fig. 9).

**Fig. 10:**
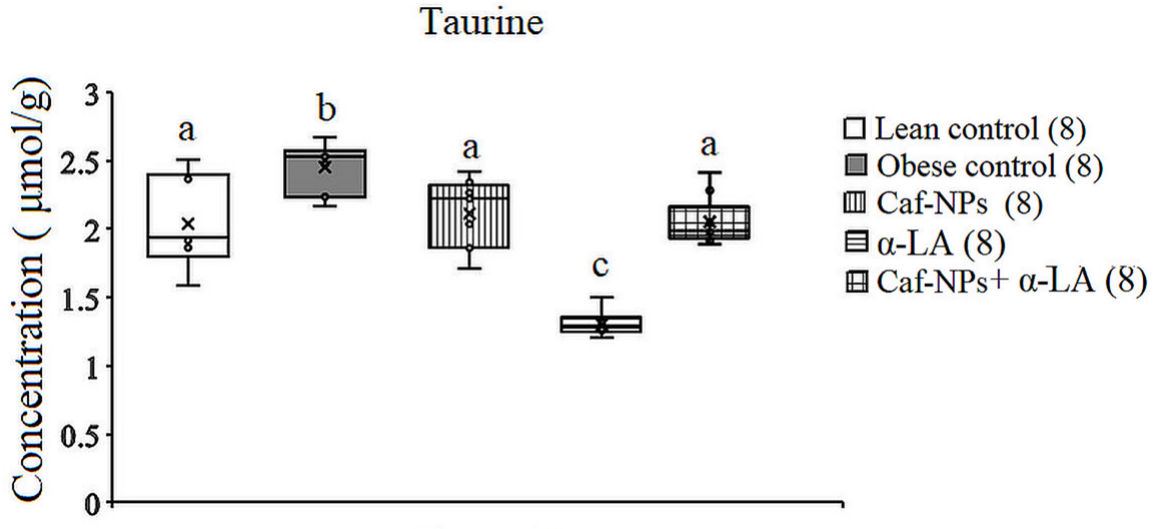
Effect of Caf-CNPs and/or α-LA on the level of taurine (µmol/g) in the hypothalamus of rat model of obesity.

The present data revealed a significant increase in taurine (*p* = 0.001, F (4,35) = 13.855) in obese rats, recording 20.93% as compared to lean control. α-LA decreased taurine (*p* = 0.001) significantly by 37.42% as compared to lean control. Caf-CNPs alone or in combination with α-LA ameliorated the increase in taurine levels induced by obesity to lean control value recording 4.2% and 1.2%, respectively (Fig. 10).

#### Leptin and Ghrelin

**Fig. 11:**
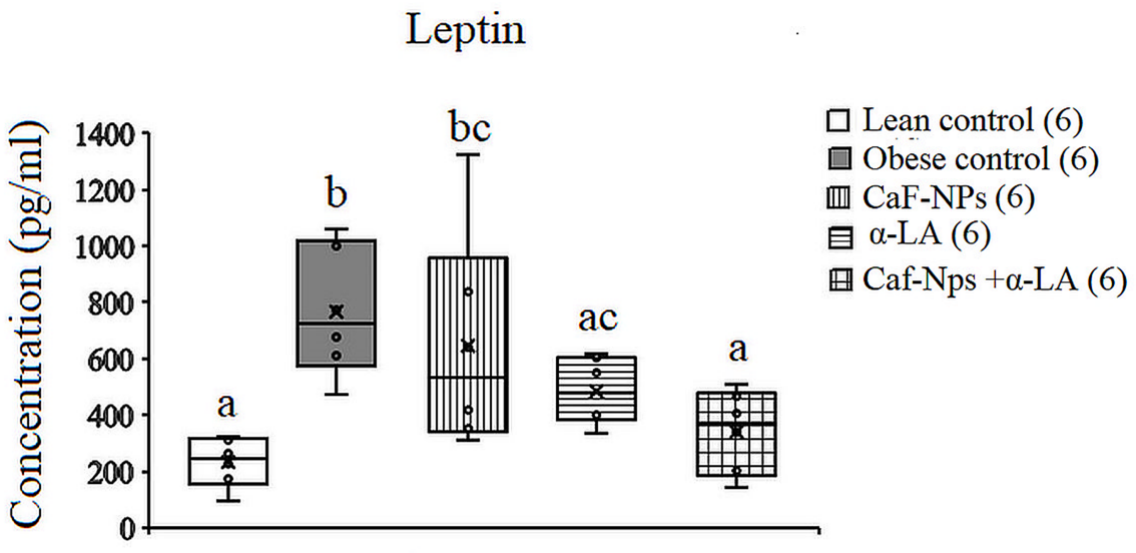
Effect of Caf-CNPs and/or α-LA on the serum level of leptin (Pg/ml) in the rat model of obesity.

The serum levels of leptin increased significantly (*p* = 0.003, F (4,25) = 5.364) in obese control rats recording 234.78% above lean control values. Although treatment of obese rats with Caf-CNPs reduced leptin levels by 182.61%, its level was still increased significantly as compared to lean control values. In the serum of obese control rats treated with α-LA alone or in combination with Caf-CNPs, leptin showed a nonsignificant increase recording 73.9% and 47.8%, respectively, as compared to lean control rats (Fig. 11).

**Fig. 12:**
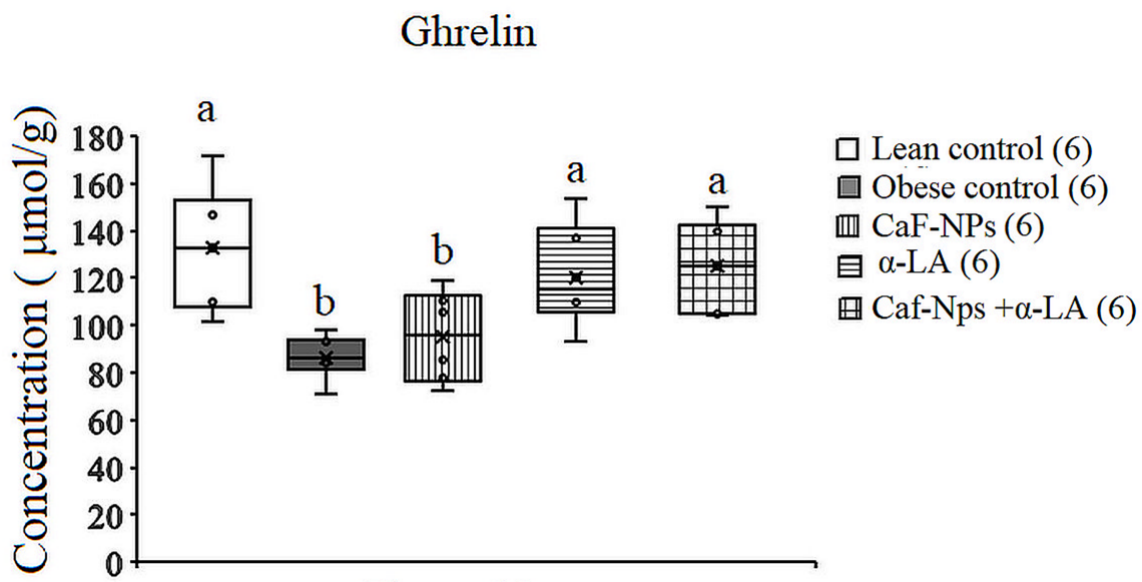
Effect of Caf-CNPs and/or α-LA on the serum level of ghrelin (Pg/ml) in the rat model of obesity.

The serum levels of ghrelin decreased significantly (*p* = 0.001, F (4,25) = 6.305) by 34.89% in obese rats as compared to lean control. This decrease continued significantly (−28.3%) after treatment of obese rats with Caf-CNPs. However, when obese control rats were treated with α-LA alone or in combination with Caf-CNPs, ghrelin levels were restored to lean control values recording 9.1% and − 5.8%, respectively (Fig. 12).

**Fig. 13:**
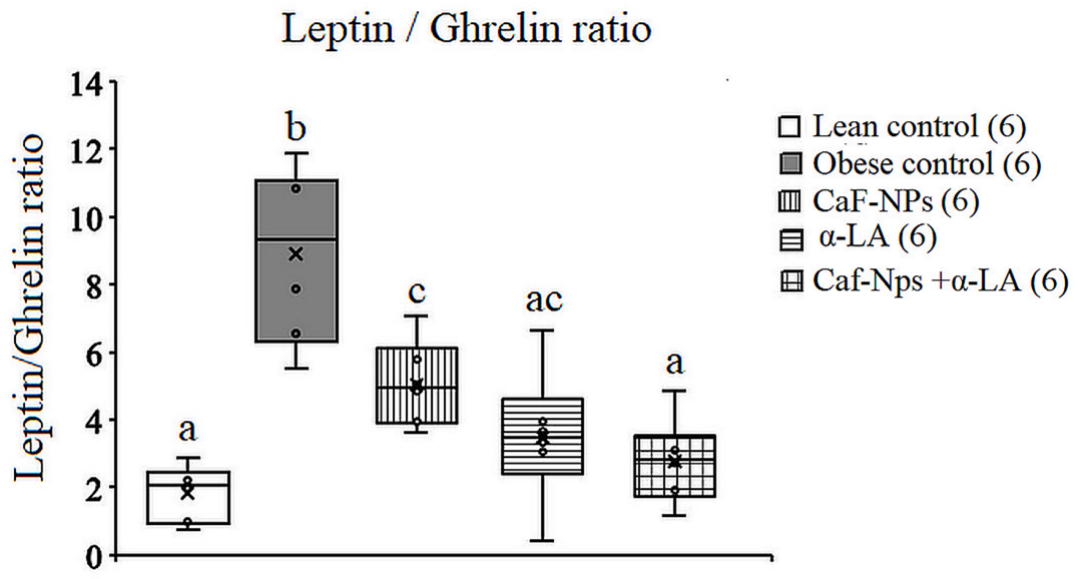
Effect of Caf-CNPs and/or α-LA on the ratio between leptin and ghrelin in the serum of rat model of obesity.

ANOVA also revealed a significant difference in the leptin/ghrelin ratio between groups. The leptin/ghrelin ratio increased significantly (*p* = 0.001, F (4,25) = 15.786) in obese rats recording 384.24% above the lean control value. Treatment of obese control rats with α-LA alone and in combination with Caf-CNPs restored the ratio to lean control values recording 91.9% and 57.2%, respectively. However, sole treatment with Caf-CNPs didn’t change the increase in leptin/ghrelin ratio (294.8%) observed in obese control rats (Fig. 13).

## Discussion

In the present study, the rat model of obesity induced by a high fat diet was used to investigate the role played by the hypothalamic excitatory (glutamate and aspartate), and inhibitory (GABA, glycine and taurine) amino acid neurotransmitters and the associated changes in the serum levels of leptin and ghrelin in obesity. Moreover, the therapeutic effects of α-LA and Caf-CNPs on the induced changes were recorded.

It has been suggested that mapping brain glutamatergic and GABAergic circuits that regulate appetite can enrich our understanding of obesity pathogenesis and therapy^23,48^.

The induction of the rat model of obesity was confirmed by calculating the BMI, which recorded 0.77 g/cm^2^ verifying the success of the obesity model according to Novelli et al.^44^. The current study focused on the hypothalamus due to its crucial role in maintaining the balance between food intake and the expenditure of energy. In the hypothalamus of obese rats, an increase in glutamate, aspartate, GABA and taurine was observed. This was associated with an increase in leptin levels and a decrease in ghrelin levels in serum.

It has been reported that mice fed a high-fat diet for 2 months showed an increase in glucose and fatty acid metabolic rate in the hypothalamus associated with hypothalamic inflammation and major metabolic changes, particularly in astrocytic metabolism and neurotransmission fluxes^49^. These findings may explain the present increase in glutamate and GABA by the switch of glutamate to GABA. as more acetyl-CoA and oxaloacetic acid (OAA) were consumed in the citrate synthetase reaction (OAA + acetyl-CoA → citrate), reducing the availability of OAA to the aspartate aminotransferase reaction (OAA + Glu ↔ Asp + α-ketoglutarate). Thus, more glutamate is directed to the glutamate decarboxylase pathway, favoring GABA synthesis and neurotransmission.

In the hypothalamus, two neuronal populations exist in the arcuate nucleus (ARC) with opposite effects on feeding behavior, namely orexigenic neuropeptides such as neuropeptide Y (NPY) and agouti-related peptide (AgRP), and anorexigenic neuropeptides including pro-opiomelanocortin (POMC)^50^. AgRP neurons are activated by glutamate resulting in an increase in food intake^51^. Moreover, an increase in glutamatergic signaling in the lateral hypothalamic area potently stimulates feeding^23^. In addition, the GABAergic inputs from the dorsal hypothalamus inhibit the anorexigenic effect of POMC leading to an increase in feeding behavior^52^. Additionally, activation of arcuate GABAergic AgRP or non-AgRP neurons leads to obesity^28^. Therefore, the present increase in hypothalamic glutamate, aspartate and GABA levels could mediate the increase in food intake leading to the development of obesity and the increase in BMI recorded in the present study.

Leptin is known as the satiety hormone and is secreted mainly by adipose tissue. The circulating levels of the hormone are proportional to the total amount of energy stored in the form of fat in the body^53^. Therefore, the present elevated leptin levels may be attributed to the increase in fats and may act on the hypothalamus by negative feedback action to control appetite and energy expenditure. In high-fat-fed mice, peripheral leptin resistance is caused by the inability of leptin to pass the blood-brain barrier^54^, whereas central leptin resistance is due to the failure to activate leptin receptors^55^. Consistent with these findings, it was reported that leptin resistance in diet-induced obesity in rats resulted in failure to stimulate hypothalamic receptors^56^. Hyperleptinemia and resistance to reduction in body mass are two main features of obesity^57^. It has been suggested that obesity in humans is caused by leptin resistance. As leptin cannot cross the BBB in spite of its elevated levels, leptin resistance develops with a sustained elevation in leptin levels^33^. This may provide an explanation for the present increase in leptin levels in obese individuals. Interestingly, the excitotoxicity induced in Alzheimer’s disease due to NMDA receptor over-excitation by glutamate can be related to obesity through leptin resistance which acts as a risk factor for neurodegenerative disorders^58^.

The present increase in hypothalamic glutamate in obese rats may also explain the damaging effect of the increased leptin. Excitotoxicity is induced as a consequence of the excessive stimulation of NMDA receptors leading to increased calcium influx and calcium overload that initiate free radicals’ production. Therefore, obesity results from the disruption in appetite and body weight control after hypothalamic structural damage^59^. Recent studies have shown that dietary fat consumption induces hypothalamic resistance to leptin and insulin, the main anorexigenic hormones, thus causing a progressive loss in the balance between food intake and thermogenesis, thereby enhancing body mass gain^60,61^.

The development of leptin resistance may lead to elevated glutamate and GABA levels which promote feeding, and this in turn may exaggerate the obesity condition.

On the other hand, ghrelin has an orexigenic effect, therefore, the present reduction in serum ghrelin concentrations in obese rats may be considered a physiological adaptation to the positive energy balance correlated with obesity. The reduced serum ghrelin level may serve to reduce the hunger and food intake due to the excessive energy stores^62^. In rodents, several studies showed that ghrelin has an important role in signaling hypothalamic centers that control feeding and caloric state^63,64^. However, the plasma concentrations of this adipogenic hormone, ghrelin, are lower in obese subjects than lean subjects, suggesting that downregulation of ghrelin occurs in obesity, probably due to the increased insulin or leptin, since a negative correlation exists between fasting plasma ghrelin levels and fasting plasma insulin and leptin levels.

In the present study, the high ratio of leptin/ghrelin observed in obese rats indicates an imbalance between leptin and ghrelin. The inverse relation between these hormones is one of the characteristics of obesity^65^ and could be attributed to the increased leptin and ghrelin resistance which elevates hypothalamic glutamate, aspartate and GABA. Moreover, an increase in taurine levels was also observed in obese rat model.

Alterations in brain taurine have been demonstrated in HFD-induced obesity models. In the hypothalamus, taurine has been reported to reduce neuropeptide Y (NPY) expression and enhance the anorexigenic effects of insulin^66^. In the periphery, it promotes insulin secretion and has anti-obesity effects^67^. The present increase in hypothalamic taurine levels in obese rats may represent a compensatory mechanism through which taurine attempts to protect neuronal cells against glutamate excitotoxicity and the metabolic syndrome.

Overall, the increased excitatory glutamate and aspartate neurotransmitters may be responsible for the damage of the hypothalamic nuclei under the effect of leptin resistance and may lead to overeating, thus exaggerating obesity and its complications.

In the present study, α-LA treatment restored the increased glutamate and aspartate, reversed the increased GABA and taurine into a significant decrease and increased glutamine. This was associated with restored serum levels of leptin and ghrelin. α-LA also reduced the BMI to 0.63 g/cm^2^ confirming its efficiency as an anti-obesity agent.

It has been reported that α-LA decreased leptin levels by reducing its secretion and gene expression^68^. α-LA has been found to increase glutamate uptake and glutamine synthase activity^69^. By this mechanism α-LA may attenuate glutamate levels in the hypothalamus by increasing its re-uptake into glial cells and its conversion to glutamine by glutamine synthase. This in turn may explain the elevated levels of glutamine in the hypothalamus of obese rats treated with α-LA. Moreover, the glutamate taken up by glial cells can be used to synthesize reduced glutathione that acts as a potent antioxidant in the brain^70^. This may contribute to the protective effect of α-LA against the free radicals resulting from overfeeding and obesity. Moreover, the ability of α-LA to restore GABA prevents its inhibitory effect on pro-opiomelanocortin, which has an anorexigenic effect. By this mechanism, α-LA suppresses appetite. The reduction in taurine levels after α-LA was expected in view of the success of α-LA in ameliorating the changes in the hypothalamic amino acid neurotransmitters and the restoration of the control-like levels of leptin and ghrelin together with the balance between them, which confirms the anti-obesity effect of α-LA.

In the present study, Caf-CNPs were also used to evaluate their effects on the changes in amino acid neurotransmitters induced in the hypothalamus of the rat model of obesity. When compared to conventional administration of native materials, nanoparticle formulations may have a number of advantages over their original form in terms of dose, effectiveness, and adverse effects^71^. Nanoparticles’ increased bioavailability makes it possible to apply smaller doses while still achieving therapeutic results. Consequently, this lowers the possibility of toxicity and adverse consequences linked to high dosages of drugs. According to Lee and Yeo^72^, the controlled release of nanoparticles may also increase their efficacy and offer a sustained medication level in the body. Caf-CNPs’ physicochemical characterization revealed that this formula had a long-lasting release that lasted for about 12 h and a high degree of stability, as shown by the zeta potential.

The present findings showed that when obese rats were treated with Caf-CNPs, a significant increase in glutamine, glutamate, aspartate, GABA and glycine was observed in the hypothalamus. However, Caf-CNPs improved the changes in leptin and ghrelin and reduced the BMI to 0.65 g/cm^2^.

However, although Caf-CNPs succeeded in reducing obesity, the changes in amino acids induced by HFD were not ameliorated. This means that Caf-CNPs could improve obesity by a mechanism other than the hypothalamic amino acid neurotransmitters.

It has been reported that the anti-obesity effect of caffeine is mediated by its inhibitory effect on the adenosine 1 receptor expressed on paraventricular neurons (PVN) in the hypothalamus^73^. Mice with overexpression of adenosine 1 (A1R) receptors in PVN showed a hyperphagic effect and an increased body weight^73^. The PVN is the target brain region of caffeine where it regulates energy metabolism through the caffeine-A1R signaling system that uses oxytocin to suppress appetite and reduce body weight by its anorexic effect^74^. In addition to its central actions, Caf-CNPs could reduce obesity by peripheral mechanisms that include reduction of lipogenesis and promotion of lipolysis^75^. Additionally, caffeine increases the release of catecholamines, which activate β-receptors and block α-receptors resulting in enhanced lipolysis and suppressed excessive fat accumulation^76^. This may explain the slight improvement in leptin. In addition to modulating metabolism through its anti-obesity, antioxidant and anti-inflammatory activities^36^, caffeine regulates obesity through the intestinal microbiota and its metabolites^37^.

The present findings demonstrated that Caf-CNPs increased glycine level. This increase may contribute to the anti-obesity effect of Caf-CNPs. Glycine is an inhibitory neurotransmitter in the spinal cord and brain^77^. It has been reported that glycine receptor agonists cause hyperpolarization of hypocretin/orexin neurons in the hypothalamus^78^. Stimulation of these neurons promotes feeding behavior^79^. Therefore, the Caf-CNPs-induced increase in hypothalamic glycine may be involved in its anti-obesity effect.

Caffeine acts as a CNS stimulant by blocking adenosine receptors and increasing the release of glutamate^80^. In the hypothalamus, adenosine activation has been found to promote the development of obesity^73^. Accordingly, Caf-CNPs could reduce obesity by blocking adenosine receptors, which in turn mediate glutamate release. This effect may explain the present increase in glutamate after Caf-CNPs treatment.

The incomplete recovery from obesity after treatment with Caf-CNPs, which is indicated by the increased leptin levels and decreased ghrelin levels, could be attributed to the increase in glutamate and GABA, which promotes feeding behavior. On the other hand, the treatment of obese rats with Caf-CNPs ameliorated the increase in the hypothalamic levels of taurine, which indicates that this amino acid may be a target of Caf-CNPs since it has been reported that the injection of caffeine and nicotine decreased cortical taurine concentration^81^.

In the current study, the co-administration of α-LA and Caf-CNPs resulted in an increase in glutamine, glutamate, GABA and glycine and restored aspartate and taurine levels. Additionally, control-like values of BMI (0.62 g/cm^2^), leptin and ghrelin were observed. These results which were observed after α-LA and Caf-CNPs co-treatment indicate a synergistic effect between the two agents. This may be due to the different mechanisms through which the two agents act to ameliorate obesity. The increased levels of glutamate and GABA could be caused by Caf-CNPs through blocking A1 receptor. However, the elevated levels of glutamine may be due to the activation of glutamine synthase by α-LA.

The elevated levels of glutamate and GABA and their promoting effect on feeding behavior may be counteracted by the elevated levels of glycine and the blocking of A1 receptors that may inhibit appetite in addition to the peripheral anti-obesity effects of Caf-CNPs. This may explain the potential anti-obesity effect observed after the combined treatment of the obese rat model with α-LA and Caf-CNPs. The recovery of the balance between leptin and ghrelin was more prominent after co-treatment with Caf-CNPs and α-LA than the sole administration of each. This may indicate the synergistic anti-obesity effect of Caf-NPs and α-LA.

## Conclusion

The present study clearly demonstrates the physiological role of the hypothalamic amino acid neurotransmitters in regulating feeding behavior and their relation to adipogenic hormones. Additionally, the changes in these neurotransmitters could be a therapeutic target for anti-obesity agents. Moreover, based on the present data, α-LA and/or Caf-CNPs could be used as safe potential non-conventional anti-obesity agents through their central and peripheral effects.

**Table 1:** BMI (g/cm^2^) of control, rat model of obesity, rat model of obesity treated with Caf-NPs and/or α-LA.

Control	Rat model of obesity	Caf-NPs	α-LA	α-LA + CAF-NPs
0.54^a^ ± 0.011	0.77^b^ ± 0.010	0.65^c^ ± 0.023	0.64^c^ ± 0.011	0.62^ac^ ± 0.015

**Table 2:** Changes in BMI (g/cm^2)^ before and after treatments in the control, obese, α-LA, Caf-CNPs and α-LA + Caf-CNPs.

Animal groups	20 weeks	BMI g/cm^2^	Daily treatment for 4 weeks	BMI g/cm^2^
Lean control rats	Fed a standard diet	0.514	Saline (0.9%)	0.54
Control obese rats	Fed HFD	0.73	Saline (0.9%)	0.77
Obese rats treated with Rat α-LA	Fed HFD	0.78	α-LA (100 mg/kg)	0.638
Obese rats treated with Rat Caf-CNPs	Fed HFD	0.79	Caf-CNPs (20 mg/kg)	0.649
Obese rats treated with α-L A+ Caf-CNPs	Fed HFD	0.791	α-L A + Caf-CNPs	0.623

## Data Availability

The datasets generated and/or analyzed during the current study are available from the corresponding author on reasonable request.
